# Mechanically evoked spike responses of pentascolopidial chordotonal organs of *Drosophila melanogaster* larvae

**DOI:** 10.1242/jeb.246197

**Published:** 2024-09-09

**Authors:** Ben Warren, Martin C. Göpfert

**Affiliations:** ^1^ Neurogenetics Group, College of Life Sciences, University of Leicester, University Road, Leicester, Leicestershire LE 7RH, UK; ^2^ Department of Cellular Neurobiology, University of Göttingen, Schwann-Schleiden Research Centre, Julia-Lermontowa-Weg 3, 37077 Göttingen, Germany

**Keywords:** Mechanoreceptor, Proprioception, Mechanotransduction

## Abstract

Mechanosensitive ensembles of neurons in insects, known as chordotonal organs (COs), function in proprioception, the detection of sound and substrate vibrations. Here, we characterized the mechanical sensitivity of the lateral pentascolopidial CO (lch5) of *Drosophila melanogaster* larvae to establish its postulated role in proprioception. We developed a physiologically realistic method to replicate proprioceptive input to lch5 by pulling the apodeme (tendon) to which the tips of the neurons attach. We found that lch5 sensory neurons respond transiently with a short latency to the velocity component of stretch displacements and the release of stretch (relaxation). In the mechanosensory mutant *inactive*, lch5 has a decreased response to mechanical stimuli and a lower overall spontaneous spike rate. Finally, we simulated the input that lch5 receives during crawling and observed spike rate changes of peristaltic body contraction. We provide a characterization of proprioceptive feedback in *D. melanogaster* larvae and firmly establish the proprioceptive function of lch5 in larval locomotion.

## Teaser

**Summary:** The spiking responses of the pentascolopidial chordotonal organ of *Drosophila* larvae to mechanical stimuli reveal a clear role in proprioception.

## INTRODUCTION

Insects are equipped with an elaborate array of mechanoreceptors, essential for responding to changes in the environment and controlling body movements. Chordotonal organs (COs) are composed of bipolar mechanosensitive neurons, which form elaborate frequency-discriminating acoustic detectors (Oldfield, 1982; Kamikouchi et al., 2009), function as proprioceptors in the context of locomotion (Usherwood et al., 1968), detect ground-borne vibration (Shaw, 1994) and serve a range of additional roles (Schnorbus, 1971; Field and Matheson, 1998). The pentameric CO of *Drosophila melanogaster* larvae (lch5) has been shown to respond to vibration and, possibly, sound (Scholz et al., 2015; Ohyama et al., 2015, 2013; Zhang et al., 2013), but characterization of lch5 responses to proprioceptive stimuli is lacking, despite its clear role in locomotion (Caldwell et al., 2003; Cheng et al., 2010; Fushiki et al., 2013; Ohyama et al., 2013; Zanini et al., 2018).

Proprioceptive COs, including the lch5 of *D. melanogaster* larvae, are typically formed of groups of scolopidia (specialized ensembles of cells containing the mechanosensory neurons), which are attached between body segments via specialist attachment cells and an apodeme (tendon-like ligament). Well-studied examples include the metathoracic femoral CO (FCO) of the locust and stick insect, which detect the relative angle between the tibia and femur. The mechanosensory neurons of the FCO alter their spike rate to a new resting position during a change in either velocity or acceleration, or a combination of these (Hofmann et al., 1985; Hofmann and Koch, 1985; Zill, 1985; Matheson, 1990; Büschges, 1994; DiCaprio et al., 2002). The neurons themselves, within any one CO, display heterogeneous responses to mechanical stimuli, known as range fractionation. For instance, any one position-sensitive neuron responds most strongly to a limited range of tibia–femur angles, but together, the FCO covers the entire range of leg angles (Hofmann et al., 1985; Zill, 1985). Similarly, any one velocity-sensitive neuron responds to a limited range of velocities, but together, the FCO codes for an extended range of velocities (Matheson, 1992). Adding to the complexity of the FCO, individual sensory neurons can be unidirectionally or bidirectionally sensitive and respond to both an increase and decrease in tension. These heterogeneous response types in multiple COs provide detailed proprioceptive feedback necessary for coordination of locomotion.

Insect larvae possess a simple array of COs essential to coordinate crawling (Caldwell et al., 2003; Fushiki et al., 2013; Titlow et al., 2014). Larvae CO neurons are accessible for electrophysiological recordings (Zhang et al., 2013; Scholz et al., 2015; Zanini et al., 2018) and fluorescent imaging (Ohyama et al., 2015), representing an attractive system to understand CO sensory input in locomotion. Our knowledge of the mechanical stimuli to which larvae CO respond is limited to responses to vibratory stimuli (Scholz et al., 2015; Ohyama et al., 2013; 2015; Zhang et al., 2013), which is thought to be a possible adaptation to detect predators (Zhang et al., 2013). Within each abdominal hemi-segment of *D. melanogaster* larvae are three singlet COs (composed of one scolopidium each) and one pentameric CO, lch5 (composed of five scolopidia). The five bipolar neurons comprising lch5 are stretched diagonally from the dorsal posterior to the lateral anterior region of each abdominal segment; ideally placed to detect contractions necessary for crawling. It is generally assumed that, in addition to detecting vibration, lch5 responds to contractions of abdominal segments and that this sensory information is necessary for coordinating locomotion (Caldwell et al., 2003; Inbal et al., 2004; Fushiki et al., 2013; Ohyama et al., 2013).

Our first aim was to characterize the spiking response of lch5 to mechanical stimuli by recording extracellular spikes from the lch5 nerve. We delivered mechanical stimuli by stretching the cap cells onto which the tips of the neurons are embedded; this is a physiologically realistic way to simulate proprioceptive stimuli. Our second aim was to characterize the response of lch5 to simulated peristaltic displacements to confirm the proprioceptive role of lch5 in locomotion.

## MATERIALS AND METHODS

### Preparation

Third instar larval *Drosophila melanogaster* Meigen 1830 (Insecta, Diptera, Drosophilidae) were pinned to an agar-coated recording chamber. Larvae were prepared using a modified fillet preparation in haemolymph-like saline containing (in mmol l^−1^): 103 NaCl, 3 KCl, 5 2-([1,3-dihydroxy-2-(hydroxymethyl)propan-2-yl]amino)ethanesulfonic acid, 10 trehalose, 10 glucose, 7 sucrose, 26 NaHCO_3_, 2 CaCl_2_, 1 NaH_2_PO_4_, 4 MgCl_2_, adjusted to pH 7.2 with KOH. For the fillet preparation, two pins (0.1×1 mm tungsten wire) were inserted through the mouth hooks and tail, pinning the larvae dorsal side up before saline was added. We used ultra-fine clipper scissors (model 15300-00, Fine Science Tools, Heidelberg, Baden-Württemberg, Germany) to cut along the dorsal midline, and a further four pins were used either side of the mouth hooks and tail pins to pin down the corners of the cuticle. All internal organs, body fat and two main tracheae were removed with fine forceps, but the muscles and nervous system were left intact. The spike rate of lch5 was temperature sensitive, changing by ∼12 spikes s^−1^ °C^−1^. Therefore, all recordings were performed at 21±1°C.

### Piezo stimulation and electrophysiology

A compact piezo-actuator and controller (Physik Instrumente GmbH, Karlsruhe, Baden-Württemberg, Germany) (P-841.10, E-709.SRG) under the control of Patchmaster software (HEKA, Lambrecht, Niedersachsen, Germany) was used to control a custom-made tungsten probe (18 mm length, 0.2 mm diameter) that was lowered and pressed on top of the long and slender cap cells of lch5, ∼30 µm distal to the cap, where the cilia are inserted, at the point where the ligament cells are narrowest. The tip of the probe had a 90 deg bend perpendicular to the field of view. We relied on friction exerted through the probe onto the cap cells to displace lch5. Piezo-actuators utilize the linear interaction between the mechanical and electrical states of piezoelectric material to alter their length based on voltage fed into them, in a linear manner. The tungsten probe was in-line and parallel with the orientation of the cap cells and ligament cells. The time for the probe to reach half maximum displacement (for the largest displacement of 7.5 µm) was 1.3 ms, with the full displacement being reached at 4.1 ms. No muscles were removed. Suction pipettes made from borosilicate glass (Science Products GmbH, Hofheim, Hesse, Germany) (GB150T-8P) pulled with a P-1000 (Sutter Instruments, Novato, CA, USA) electrode puller to a tip diameter of ∼4 µm and filled with saline described above were used to suck the lch5 nerve (∼20 µm) into the pipette. After this, gentle suction was used to attach the pipette to the sheath surrounding the somata of lch5. Spontaneous spike frequency was measured directly after the recording pipette and stimulus probe were in position. A HEKA patch-clamp amplifier (EPC 10 USB) under the control of Patchmaster software (HEKA) was used in voltage-clamp mode and data were filtered online with an analog four-pole low-pass Bessel filter at 2.9 kHz. Data were sampled at 20 kHz. Voltage-clamp mode increases the extracellular signal by injecting a counter-current to increase the electrochemical gradient across the membrane, but the extracellular space is not voltage-clamped, as occurs in conventional whole-cell voltage clamp.

### Microscopy, charge-coupled device (CCD) imaging and laser Doppler vibrometry

**Fig. 1.**
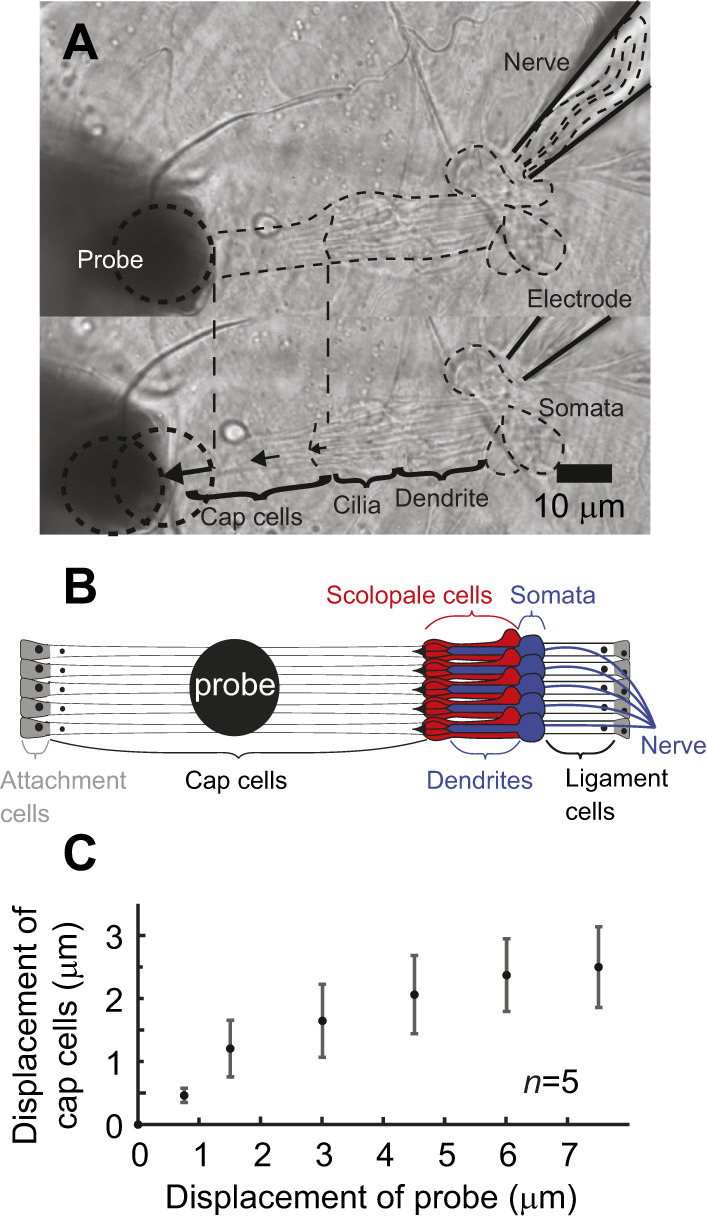
**Mechanical stimulation of lch5 and relative displacement of probe and cap cells.** (A) Displacement of lch5 with tungsten probe visualized with DIC microscopy. (B) Displacement (±s.d.) of tungsten probe tip when *in situ*, when pressed against the cap cells to impose step stimuli of five separate lch5s from five separate larvae. (C) Cap cell displacement was measured at the proximal end, where the cilia of the auditory neurons are inserted.

An Examiner D.1 microscope (Zeiss, Oberkochen, Baden-Württemberg, Germany) with a 63× objective (Zeiss, 424516-9041) and differential interference contrast (DIC) slider (Zeiss, 426961) and an AxioCam MRm (Zeiss, 1388×1040 pixels) under the control of Axiovision (Zeiss V.4.8.2.0) were used to image the probe and neuron displacements. A laser Doppler vibrometer (Polytec, PSV-400, Harpenden, Hertfordshire, UK) was used to confirm displacements of the probe. Sinusoidal stimuli delivered to the probe delivered reliable amplitude up to between 60 and 100 Hz. We used a white noise stimulus to displace the tungsten probe and record its frequency response. The tungsten probe exhibited an almost flat frequency response within the frequencies used (Fig. 1A).

### Inactive mutants

Canton S larvae were used as wild-type strains. *iav*^1^ mutants as described previously (Gong et al., 2004) were kindly provided by Maurice Kernan (Stony Brook University, Department of Neurobiology and Behavior, USA).

### Data analysis and statistics

Igor software (Wavemetrics, Lake Oswego, OR, USA) (version 6.3.2.3) was used to high-pass filter the data (Finite Impulse Response Filter, end of reject band, start of pass band 500, 555 Hz). Spike 2 software (Cambridge Electronic Design, Cambridge, UK) (version 7) was used for spike sorting. The time window was set to 0.4 ms before and after the peak amplitude of spikes. The threshold of spike detection was 20 pA. Automatic template generation was used and spikes were classified as the same when within 10% of the same amplitude. Statistical tests were computed in Microsoft Excel and all error bars are standard deviation. Linear models (LMs), also known as *t*-tests, were used for pairwise comparisons; *t*-values are reported to indicate the extent of the change. *P*-values are reported to indicate the probability of the data, or more extreme data, if the null hypothesis [i.e. that displacement of lch5 does not affect spike rate (see Fig. 3) or that there is no change in spike rate directly after ramp onset (see Fig. 4)] is correct.

## RESULTS

### Mechanical stimulation

We applied mechanical stimuli to lch5 by a piezo-actuator-coupled tungsten probe lowered onto the cap cells ∼30 µm distal to the cap, where the cilia of the neurons are embedded in abdominal segments A2–A5 (Fig. 1A). The probe pushed through overlying muscles to displace in-line and parallel with the long axis of lch5. We measured displacements of the probe and the cap cells optically using DIC optics of the microscope and a CCD camera (Fig. 1A). There was a linear Hookian relationship between the probe and cap cell movements, though for larger displacements the cap cells moved relatively less, presumably as the friction of the probe onto the lch5 cap cells was overcome and the probe slipped along its site of contact at the cap cell (Fig. 1B). The constant pressure of the probe on the cap cell, necessary to displace lch5, could shift the resting tension of lch5. However, we measured both increases and decreases in lch5 spike rate to positive and negative displacements of the probe, suggesting we have not exceeded the physiological range of lch5. The range of displacements delivered to lch5 was limited to 15 µm, which is comparable to the 462±80 µm length of the lch5 from its two attachment sites and an approximate length change of lch5 of 77 µm, or 20% (Hassan et al., 2019). Probe displacements beyond 15 µm resulted in the nerve of lch5 being pulled out of the recording electrode. Occasionally, abdominal muscles contracted, as occurs in locomotion, and lch5 bent from straight to S-shaped. This constitutive contracted state rendered spike recordings from lch5 impossible. All recordings of lch5 were made in the relaxed non-contracted state.

### Spontaneous spiking properties of lch5

**Fig. 2.**
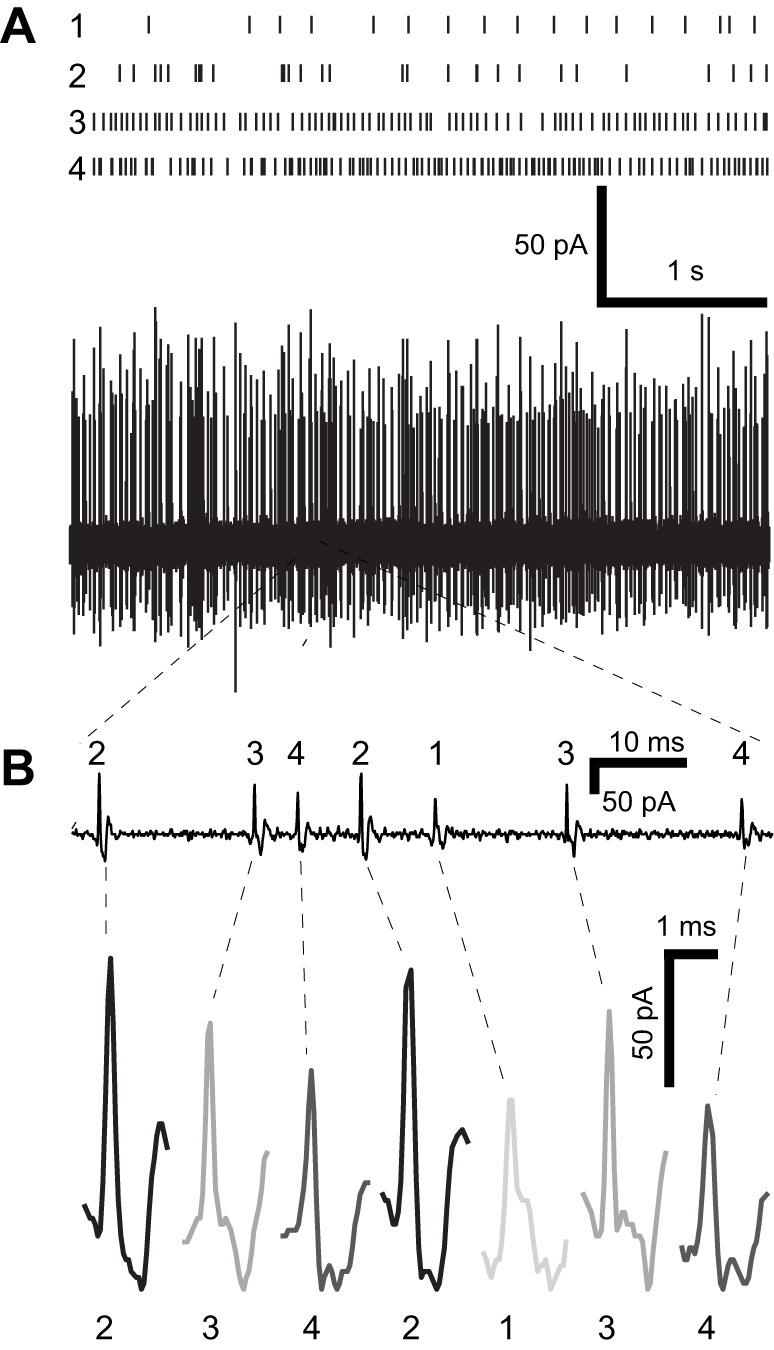
**Extracellular recording of spontaneous spiking activity from an example lch5 and spike sorting.** (A) Extracellular currents recorded from lch5 nerve (middle trace), with raster plot of four spike types (1–4) and (B) zoom in of time trace (bottom) showing the four spike types.

Spontaneous spikes were recorded at an average frequency of 46.6±15.3 spikes s^−1^ (*n*=20) from the lch5 nerve. A spike-sorting algorithm was used to identify individual spike-forms (units) from lch5 (Fig. 2A). The spike frequency of distinguishable spike-forms had a range of 1.5 to 78.4 Hz with an average of 24.6±23.2 Hz (*n*=34 neurons from 11 lch5 neurons). The number of distinguishable spike-forms ranged from one to four, representing four of the five sensory neurons in lch5 (Fig. 2B). It is possible that spike-forms from multiple neurons were indistinguishable from the spike-sorting algorithm and that the number of neurons spontaneously spiking was underestimated by our analysis. All remaining analysis uses the pooled response of all lch5 units.

### Spike latency and response to step stimuli

**Fig. 3.**
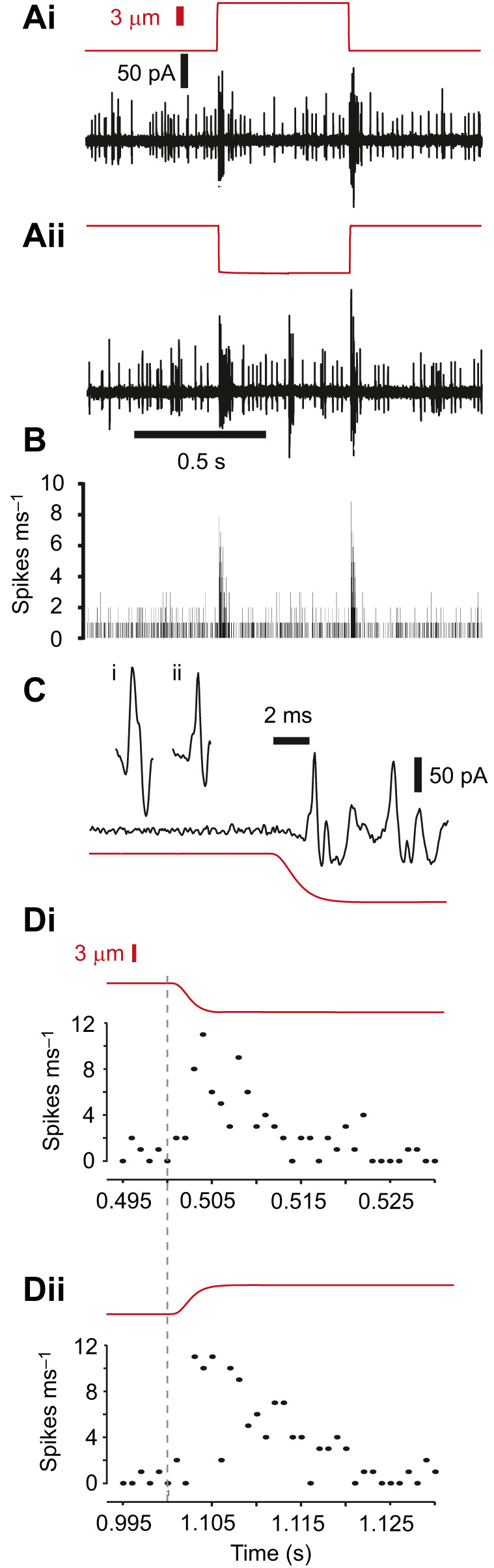
**Latency of spikes recorded from lch5 in response to step displacements.** (A) Extracellular recording from lch5 nerve (black) in response to a (Ai) positive (push) step displacement (red) and (Aii) negative (pull) displacement. (B) Histogram of seven consecutive recordings from the same lch5 for pull displacements with 1 ms bins. A threshold of 20 pA was used to count spikes. (C) Extracellular recording and step displacement on expanded time axis showing (Ci) a step elicited spike and (Cii) a spontaneous spike. Red trace is sensory feedback from the piezo actuator. (D) Pooled spike responses from eight separate lch5s on an expanded time-axis in response to (Di) push and (Dii) pull 7.5 µm displacements.

We measured the combined spiking responses of lch5 in response to step displacements of 7.5 µm amplitude (away from lch5, defined as negative displacement). lch5 responded with spikes (Fig. 3A,B) within 2.36±0.68 ms (*n*=10) (Fig. 3C). It is likely that we underestimated the spike latency owing to delays in probe movement (time to half maximum displacement was 1.3 ms for the largest displacement), and viscous effects of the tissue and saline. In order to determine the short-term duration of response to a step stimulus, we counted spikes in 1 ms bins. Spike response significantly increased during the 10 ms after displacement (Fig. 3B) (LM: *t*_20_=6.95, *P*<4×10^−7^, comparing ten 1 ms bins before and after displacement). These spikes were of similar amplitude and waveform as spontaneous spikes, suggesting they were not artifacts (Fig. 3i,ii). We pooled the responses of eight separate lch5 neurons in response to push and pull displacements and plotted responses in the first 30 ms to analyse whether lch5 had adapting tonic units. The number of spikes decreased to spontaneous levels within 15 ms. The number of spikes within any 1 ms time bin was 11 (out of a possible 40 units from eight lch5s), but the total number of spikes for push and pull displacements was 62 and 90, respectively, suggesting that individual neurons spiked more than once during a single step displacement.

### Lch5 neurons are velocity sensitive

**Fig. 4.**
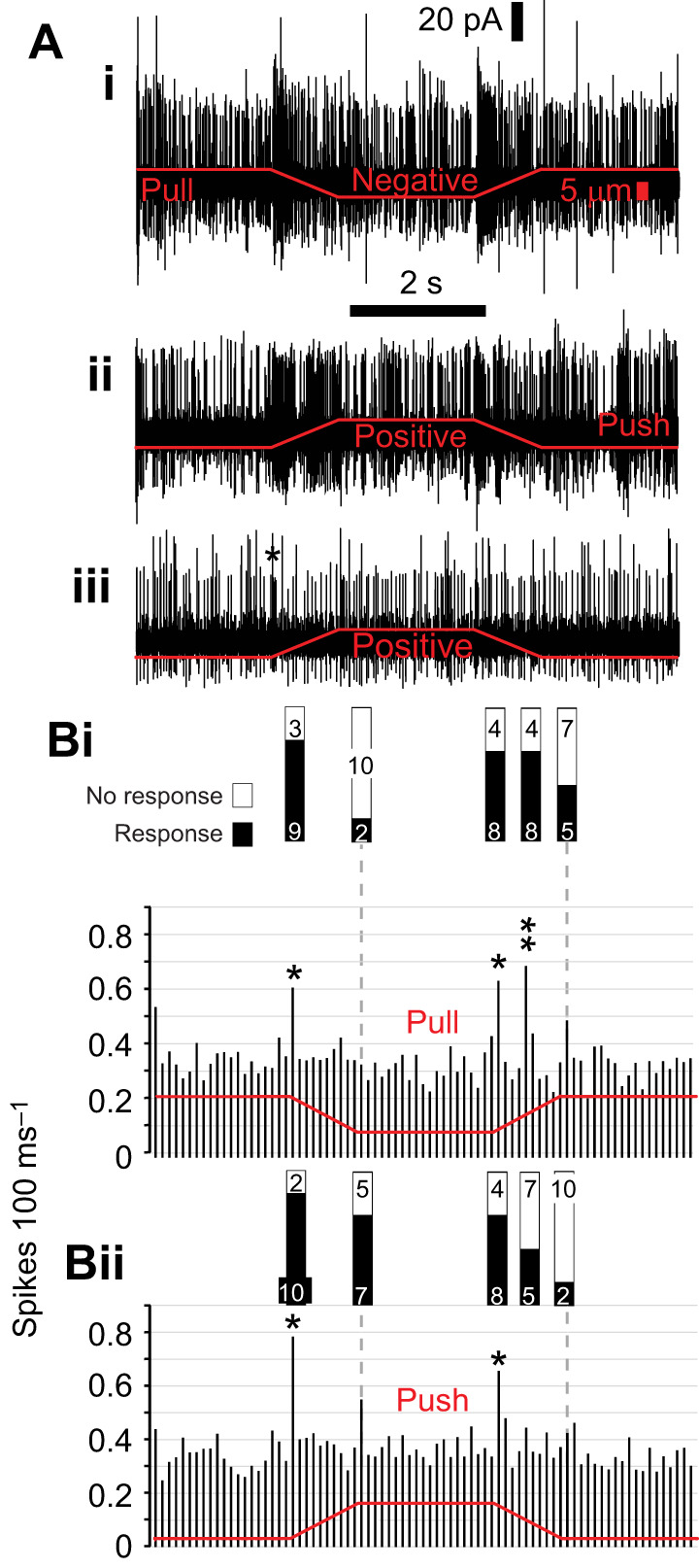
**Extracellular spike responses of lch5 to ramp and hold displacements when pulling and pushing the cap cells.** (A) Extracellular recording from an lch5 nerve in response to a (Ai) pulling (negative) ramp and hold displacements and a (Aii, Aiii) pushing (positive) direction. (B) Histogram of spike responses of 12 lch5 nerves from 10 larvae with 100 ms bins to negative (Bi) and positive (Bii) displacements. Asterisk donates significant increases in spike rate. The proportion of all 12 lch5 nerves from 10 larvae responding is indicated above by black infill to the bars. Responses were also common on the release of tension, donated by double asterisk. Data for individual responses and histograms are available in Figs S1 and S2 (for Fig. 4Bi and 4Bii).

To characterize proprioceptive-like lch5 responses, we used ramp-and-hold displacements in both directions [away from (negative) and toward (positive) lch5] with 1 s rise and fall times (Fig. 4Ai,ii). Ramp-and-hold displacements are an established method for identifying position-, velocity-, acceleration- and bidirectionally sensitive proprioceptive neurons. Spike response increased typically at ramp onset for both push and pull mechanical stimulation (Fig. 4Ai,ii). The majority of mechanically elicited responses were confined to either the pull or push ramps (Fig. 4Aiii, asterisk). Spikes were pooled into 100 ms bins to capture the duration of neuron responses. The number of spikes for each 100 ms bin was then normalized to the largest number of spikes in any one bin for each lch5 to visualize responses (Fig. 4Bi,ii). Spike response typically increased for ramp onset but less frequency for ramp offset. To identify lch5 neurons that responded to either positive or negative ramp stimulations, significant increases in spike rate were classified if the number of spikes in the 100 ms bins (indicated with asterisks) were at least three times the standard deviation of the mean spike number of the remaining 100 ms bins (i.e. all remaining bins without an asterisk). We set a threshold of three times the standard deviation to account for the variation (26–102 Hz) in the spontaneous spike rate between individual lch5 neurons. The proportion of 12 lch5 neurons responding to each phase of the ramp-and-hold mechanical stimulus ranged from 10 of 12 for ramp onset for push stimulation down to 2 of 12 for ramp offset for push and pull stimulation (Fig. 4Bii). Responses from individuals are contained in Figs S1 and S2 (for Fig. 4Bi and 4Bii).

A significant increase in spike rate occurred midway through the off ramp for negative displacements (Fig. 4B, question mark) (LM: *t*_76_=6.84, *P*=9×10^−10^, comparing two 100 ms bins of response with remaining 100 ms bins). Displacement-correlated responses were not limited to 100 ms following ramp onset of step-and-hold stimuli (Fig. 4B, arrow). There was a significant increase in spike rate with ramp offset for negative displacements (Fig. 4B, arrow) (LM: *t*_76_=2.45, *P*=0.008, comparing two 100 ms bins of response with remaining 100 ms bins).

### Velocity/acceleration threshold of lch5 neurons

**Fig. 5.**
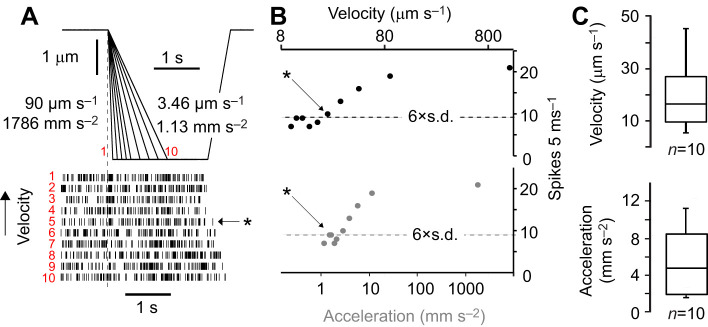
**Experimental protocol to determine the velocity/acceleration threshold of lch5.** (A) Displacement of tungsten probe and raster plot of spikes from one lch5 to different velocities and accelerations of displacement indicated by red numbers from 1 to 10 (red number 1 is 90 µm s^−1^ and 1786 mm s^−2^ and red number 10 is 3.46 µm s^−1^ and to 1.13 mm s^−2^). (B) Plot displaying the spikes per 5 ms bin of one lch5 in response to different ramp velocities and accelerations, asterisk (also in A) corresponds to threshold velocity/acceleration, when the number of spikes is above six times the standard deviation (i.e. significantly above noise). (C) Quantification of the velocity and acceleration thresholds found for 10 separate lch5s in four larvae.

We stretched lch5 at different velocities but with the same displacement (Fig. 5A). This protocol determined the threshold velocity/acceleration necessary to elicit a significant increase in spiking of lch5. Histograms with 5 ms bins were generated from spiking responses in order to detect the time-course of spikes for different velocities. When the number of spikes per bin was more than six times above the standard deviation (i.e. significantly above the spontaneous spiking rate) of all bins over the 4 s recording period, we classified this as a response above threshold (Fig. 5B). There was considerable spread of velocity and acceleration thresholds from 5–45 and 1.6–11.2 µm s^−2^ (Fig. 5C). The increase in spiking rate was transient in response to the velocity/acceleration component of mechanical stimuli, i.e. there was no tonic spiking. Such transient responses at the beginning of a velocity stimulus are also indicative of acceleration-sensitive units, although acceleration-sensitive units responses are usually limited to only one spike in femoral COs (Matheson, 1990; Hofmann and Koch, 1985; Büschges, 1994).

### Lch5 responds to the dynamic components of crawling

**Fig. 6.**
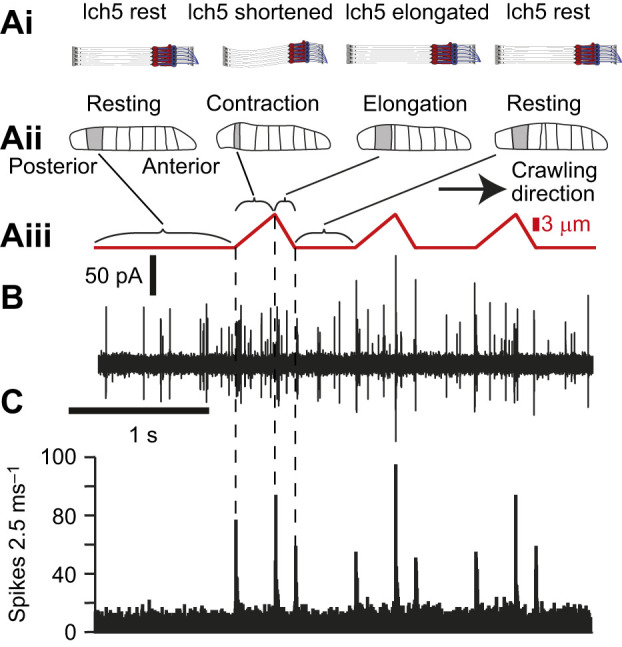
**Spike responses to simulated peristaltic contractions.** (Ai) During muscle contractions of a segment lch5 is compressed and stretched before returning to the ‘resting’ state. (Aii) Each segment has three distinct phases for crawling (grey). (Aiii) Simulated crawling stimulation from probe in red. (B) Spike responses of lch5 to displacements mimicking three cycles of peristaltic contractions. (C) Histogram of spike responses from lch5 using 2.5 ms bins of 10–20 repetitions (of the protocol shown in Aiii) in five separate lch5s in five separate larvae.

To provide physiologically realistic proprioceptive input to lch5, we used a pattern of probe movement that mimicked movements measured in the muscles of *D. melanogaster* larvae (Heckscher et al., 2012) (Fig. 6B, red trace). We repeated the crawling stimulus every 1 s as this is a typical period for *D. melanogaster* larvae crawling contractions (Wang et al., 1997; Saraswati et al., 2004). The lch5 responded transiently and consistently to the onset and offset of movement. These three phases of the cycle correspond to the (1) start of muscle contraction, (2) change to muscle elongation and (3) when muscles stop contractions (Fig. 6).

### Inactive mutant phenotypes

**Fig. 7.**
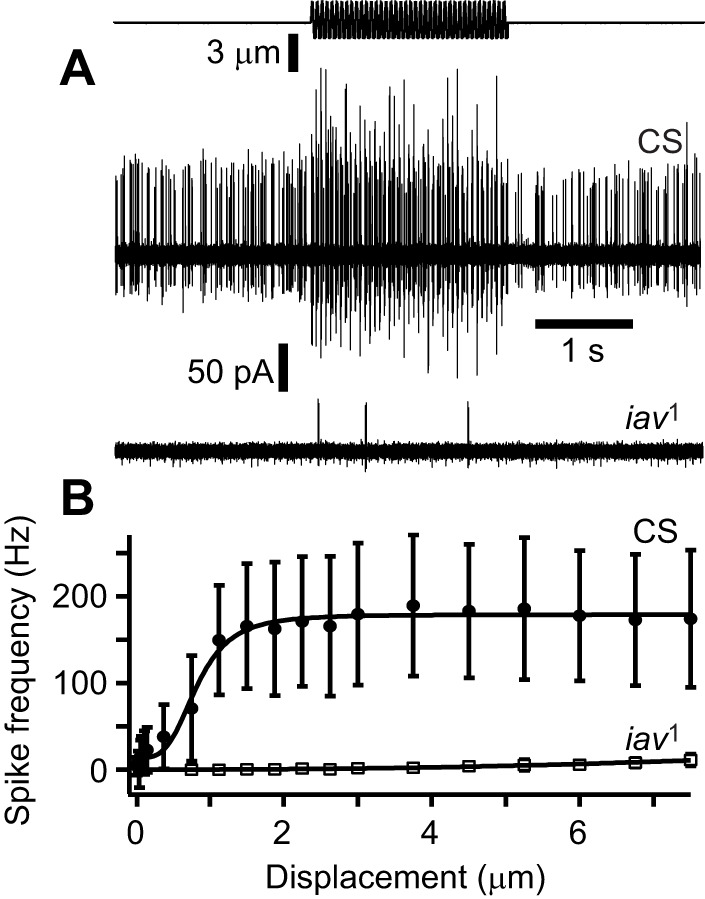
**Response of *inactive* and Canton S control lch5 to 20 Hz sinusoidal stimulation of different amplitudes.** (A) Stimulus displacement (top) and extracellular spike responses of Canton S (CS) and *iav^1^*. (B) Quantification of spike frequency increase ±s.d. to different amplitudes of Canton S (5 larvae, 12 lch5) and *iav^1^* (8 larvae, 11 lch5). Spikes with an amplitude over 20 pA were counted.

We investigated the spiking properties of lch5 in the mutant larvae carrying a null allele, *iav^1^*, of the gene *inactive* (*iav*). The Inactive protein forms a heteromeric transient receptor potential vanilloid (TRPV) channel with Nanchung in the proximal region of chordotonal neuron cilia, and its loss severely affects the mechanical properties of the adult fly's Johnston's (chordotonal) organ and sound-evoked compound potentials recorded from Johnston's organ neurons (Gong et al., 2004; Göpfert et al., 2006). lch5 of *iav^1^* null mutants exhibited a greatly reduced spontaneous firing rate (4.2±7.2 Hz, *n*=11) compared with Canton S wild-type controls (46.6±15.3 Hz, *n*=20) (Fig. 7A). The spiking responses of *iav^1^* mutants to 20 Hz sinusoidal stimulation were also significantly attenuated across the range of tested displacements (Fig. 7B).

## DISCUSSION

The genetic amenability of *D. melanogaster* has made it an excellent model organism; however, its tiny size has hampered efforts to investigate electrophysiological properties of individual sensory cells in COs. In contrast, intracellular recordings have been performed in many proprioceptive COs in other insects, which lack the genetic tools available in *D. melanogaster* to generate CO-specific mutations (Hofmann et al., 1985; Zill, 1985; Field and Pflüger, 1989; Matheson, 1990; Büschges, 1994). Extracellular recordings from COs in *D. melanogaster* larvae have previously been performed (Zhang et al., 2013; Ohyama et al., 2015; Scholz et al., 2015) but investigation of their physiology has been limited to responses to vibrational stimuli. This study investigates spiking response of the lateral pentascolopidial CO (lch5) to step/ramp stimuli using extracellular recordings. We also measured quantitative differences in spike responses between Canton S controls and *iav^1^* mutants.

Previous findings support a simple viscoelastic cap cell apodeme in *D. melanogaster* larvae with an elastic limit that is overdamped (Prahlad et al., 2017). Such biophysical properties are assumed to be largely determined by the stiffness of actin cytoskeleton in the cap cells, which could be regulated by myosin motors (Prahlad et al., 2017). The elasticity of lch5 over an extended range ensures proprioceptive feedback over a large dynamic range. Here, measurements of lch5 displacements have been inferred through probe position, but fast imaging could be used to track cap cell position directly, providing a direct measure of lch5 stimulation. Compared with a simple viscoelastic property of the cap cell apodeme, the anatomy of CO apodemes in adult insects is more complex (Shelton et al., 1992; Nowel et al., 1995). For instance, the apodeme of the metathoracic FCO of the locust has multiple ligaments that are sequentially stretched and recruited and could give rise to range fractionation (Field, 1991; Shelton et al., 1992). In the lch5 of *D. melanogaster* larvae, all cap cells attach to the same points and when inspected through DIC microscopy have a homogeneous appearance.

When the tungsten probe remained stationary, individual sensory neurons of lch5 spiked at a rate of 24.6±23.2 Hz. Spontaneous spiking is a feature typical of proprioceptive COs in insects which respond to mechanical stimulation by altering their spike rate above or below their spontaneous rate (Hofmann et al., 1985; Hofmann and Koch, 1985; Zill, 1985; Field and Pflüger, 1989; Matheson, 1990; Büschges, 1994; DiCaprio et al., 2002). The lch5 of *D. melanogaster* larvae responds to mechanical step stimuli with a short-latency change in the spiking rate of 2.36±0.68 ms (*n*=11). This latency is similar to acceleration-sensitive receptors in the stick insect FCO (Hofmann and Koch, 1985), but lies above that measured in acoustic responses of the Johnston's organ of *D. melanogaster* (∼500 µs latency) (Albert et al., 2007). Latencies here are limited by the response time of the piezo-actuator and probe, but could also be limited by the time taken for the apodeme to stretch to a threshold amount. The muscles through which the probe presses onto lch5 could add to the delay, as probe displacements would first deform and stretch overlaying muscles before displacing lch5 underneath. Previous work (Prahlad et al., 2017) shows that the long, slender cap cells of lch5 behave as a viscoelastic material, but sudden movements, such as step stimuli used here, may cause a delay in stretching of the apodeme as viscous effects momentarily overcome elasticity, and also owing to indirect connection of the probe to the cap cells and possible delay in uptaking cap-cell slack after a compression (push) displacement.

The sensory neurons of lch5 fire transiently in response to a change in displacement. Some neurons must fire more than once in response to a step stimulus, as the total number of spikes responding within the first 15 ms to cap cell pull was, on average, 11, but would be 5 if each individual neurons spiked only once. For crawling responses, lch5 spikes were elicited within the first 15–20 ms of the change in displacement with an average of 1.5 spikes over this period. This suggest that some neurons are spiking more than once and adapting, but that most neurons fire only once, even to a prolonged >100 ms change in displacement. The sensory neurons of lch5 are either responding transiently to velocity, with an average threshold of 19.1±7.2 µm s^−1^, or are acceleration-sensitive, with an average threshold of 4.61±2.9 µm s^−2^. Either way, the transient-phasic nature of these responses indicates that lch5 senses and provides feedback for dynamic movements and that the distance between body segments is not signalled by lch5. As we were unable to match the large displacements during normal crawling, we cannot rule out tonic responses of lch5 that code for displacement. The other main type of sensory neuron, multidendritic neurons that completely tile the body wall, seem well placed to provide feedback on the longitudinal state of body segments (Grueber et al., 2002). In particular, type I multidendritic and bipolar neurons, the dendrites of which span the width and length of each segment, appear to provide most of the sensory feedback necessary for normal locomotion (Hughes and Thomas, 2007). Their presumed ability to signal length in body segments may make position-sensitive CO sensory neuron responses redundant in soft-bodied larvae, an idea first suggested by Hughes and Thomas (2007).

Although peristaltic contractions necessary for larval locomotion are still present when sensory input is disrupted, sensory feedback is necessary for altering central pattern generator output and for normal locomotive behaviour (Caldwell et al., 2003; Hughes and Thomas, 2007; Song et al., 2007; Fushiki et al., 2013; Titlow et al., 2014). A goal in the field is to identify the sensory neurons necessary and sufficient for providing sensory feedback for locomotion. The respective roles of CO neurons and multidendritic neurons remain contested. Silencing these neuron types selectively by temperature-sensitive *shibire* resulted in different effects on crawling speed. Whilst two studies found a decrease in crawling speed when only multidendritic neurons were inhibited (Hughes and Thomas, 2007; Song et al., 2007), another found that larval crawling speed was decreased by inhibiting only COs (Fushiki et al., 2013). A further study using CO mutants found a decreased speed of crawling compared with controls (Caldwell et al., 2003).

Hughes and Thomas (2007) have suggested a ‘mission accomplished’ model of sensory feedback based on inferences of how multidendritic neurons operate in adult insects. The mission accomplished model postulates that bipolar neurons and class I multidendritic neurons increase or decrease in their spike rate to signal a successful muscle contraction to the central nervous system. This coordinates relaxation of muscles in that segment and contraction of muscles in the next, more anterior segment. Our simulation of peristaltic contractions delivered to lch5 revealed that a discrete number of spikes closely follows the start, middle and end of a simulated body contraction (Fig. 6). Thus, signals from lch5 could provide a ‘mission accomplished’ signal at the end of each of these three phases of a crawling cycle, which could be integrated with sensory input from multidendritic neurons to influence motor output.

The COs in *D. melanogaster* larvae have been shown with calcium imaging and focal extracellular recordings to be sensitive to vibration (Ohyama et al., 2013, 2015; Zhang et al., 2013; Scholz et al., 2015). Furthermore, detection of sound is thought to be important for *D. melanogaster* larvae to avoid predators (Zhang et al., 2013). We were unable to elicit responses of lch5 to sound of various frequencies from a loudspeaker, even at high sound pressure levels >100 dB SPL, which suggests that lch5 is fundamentally vibration-sensitive and not sound-sensitive. A parallel proprioceptive role of larval COs in *D. melanogaster* is also suggested by the study of locomotion phenotypes where COs are genetically compromised (Caldwell et al., 2003; Wu et al., 2011; Zhou et al., 2012; Fushiki et al., 2013; Zhang et al., 2013; Zanini et al., 2018), and also because lch5 spans across body segments (Klein et al., 2010). Proprioceptive FCOs in insects that have evolved into dedicated vibration receptors (for example, the subgenual organ of the cockroach leg) bear morphological specializations, whereby it no longer spans adjacent body segments (Schnorbus, 1971; Moran and Rowley, 1975; Shaw, 1994). The present study provides *in vivo* extracellular recordings of lch5 showing responses to simulated peristaltic contractions. The lch5 appears to serve a dual sensory role: responding to vibration and proprioceptive cues. To investigate the extent to which lch5 is a vibration/proprioceptive sensory organ, both types of stimuli need to be delivered in the same experimental setup. We were unable to investigate vibrational responses here as our dedicated proprioceptive setup was unable to stimulate lch5 at frequencies over 100 Hz.

To date, investigations of *D. melanogaster* larval locomotion have used genetic tools combined with behavioural analysis. Systematic physiological analysis of sensory neurons in response to proprioceptive stimuli is now needed to further identify the sensory neurons necessary for proprioceptive feedback and to start to build realistic models. Such multimodal analysis certainly seems warranted as signals from different sensory neuron types are integrated and converge in the nervous system of *D. melanogaster* larvae (Ohyama et al., 2015). Quantitative differences in sensory neuron function of mutations affecting COs in larvae have been shown with calcium imaging (Zhang et al., 2013). We now add another level of analysis by finding quantitative differences in the spontaneous spike rate and spike response to sinusoidal stimuli of sensory neurons of *iav^1^* mutants when compared with control larvae. Such findings set the stage for using the ‘simpler’ locomotory system of *D. melanogaster* larvae to understand how COs contribute to proprioceptive input.

## Supplementary Material

10.1242/jexbio.246197_sup1 Supplementary information
