# Supplementary material for: Mechanically evoked spike responses of pentascolopidial chordotonal organs of Drosophila melanogaster larvae
Source: J Exp Biol. 2024 Sep 9;227(17):jeb246197. doi: 10.1242/jeb.246197 (PMC11418168; doi:10.1242/jeb.246197)
Supplement: Supplementary information [file jexbio-227-246197-s1.pdf]

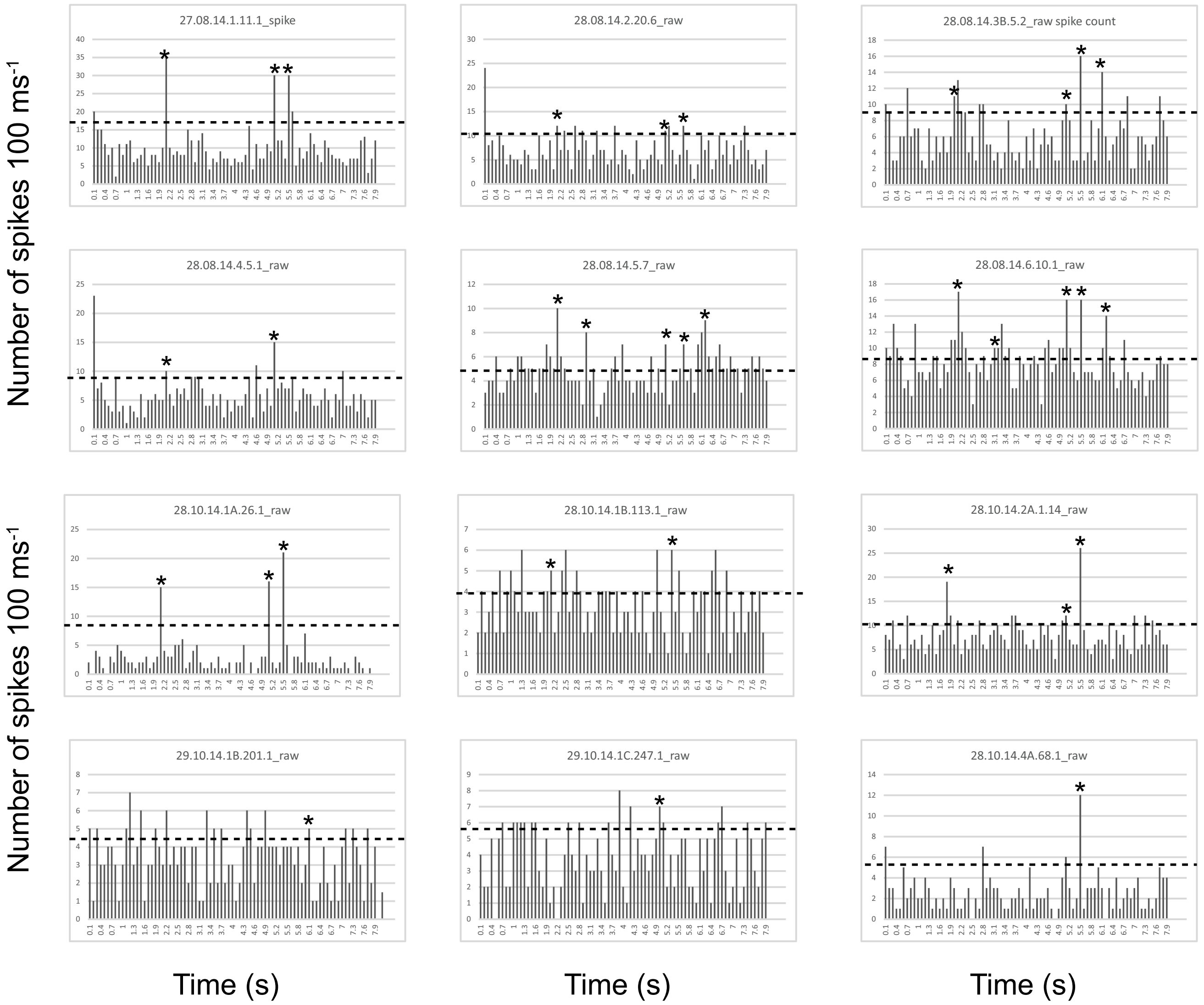

Fig. S1. The responses of 12 individual lch5s to a pull stimulation that make up Fig. 4 Bi. The threshold for responses was determined at 3XSD of the number of spikes of all the 100 ms bins in the recording (black dashed line). Asterix mark the significant responses at five time points: start of first ramp, end of first ramp, start of return ramp, end of return ramp and the midpoint of the return ramp at times 2.1, 3.1, 5.1, 6.1 and 5.6.

Journal of Experimental Biology • Supplementary information

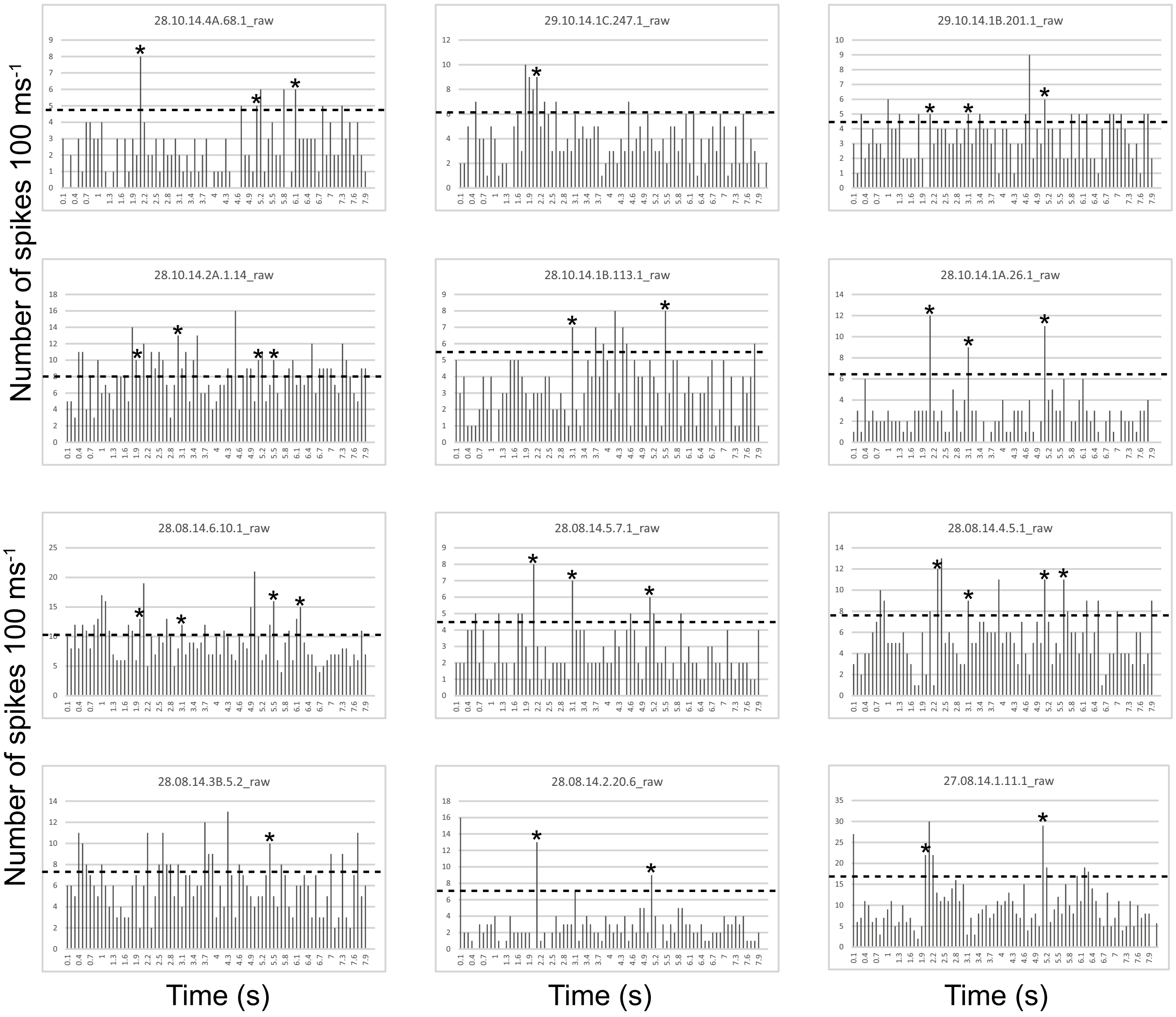

Fig. S2. The responses of 12 individual lch5s to a pull stimulation that make up Fig. 4 Bii. The threshold for responses was determined at 3XSD of the number of spikes of all the 100 ms bins in the recording (black dashed line). Asterix mark the significant responses at five time points: start of first ramp, end of first ramp, start of return ramp, end-of return ramp and the midpoint of the return ramp at times 2.1, 3.1, 5. 1, 6.1 and 5.6.

Journal of Experimental Biology • Supplementary information
